# Intelligent Chiral Sensing Based on Supramolecular and Interfacial Concepts

**DOI:** 10.3390/s100706796

**Published:** 2010-07-13

**Authors:** Katsuhiko Ariga, Gary J. Richards, Shinsuke Ishihara, Hironori Izawa, Jonathan P. Hill

**Affiliations:** 1 World Premier International (WPI) Research Center for Materials Nanoarchitectonics (MANA), National Institute for Materials Science (NIMS), 1-1 Namiki, Tsukuba 305-0044, Japan; 2 Japan Science and Technology Agency, CREST, 1-1 Namiki, Tsukuba 305-0044, Japan

**Keywords:** chiral sensing, supramolecular chemistry, interface, molecular recognition, nanotechnology, nanomaterial

## Abstract

Of the known intelligently-operating systems, the majority can undoubtedly be classed as being of biological origin. One of the notable differences between biological and artificial systems is the important fact that biological materials consist mostly of chiral molecules. While most biochemical processes routinely discriminate chiral molecules, differentiation between chiral molecules in artificial systems is currently one of the challenging subjects in the field of molecular recognition. Therefore, one of the important challenges for intelligent man-made sensors is to prepare a sensing system that can discriminate chiral molecules. Because intermolecular interactions and detection at surfaces are respectively parts of supramolecular chemistry and interfacial science, chiral sensing based on supramolecular and interfacial concepts is a significant topic. In this review, we briefly summarize recent advances in these fields, including supramolecular hosts for color detection on chiral sensing, indicator-displacement assays, kinetic resolution in supramolecular reactions with analyses by mass spectrometry, use of chiral shape-defined polymers, such as dynamic helical polymers, molecular imprinting, thin films on surfaces of devices such as QCM, functional electrodes, FET, and SPR, the combined technique of magnetic resonance imaging and immunoassay, and chiral detection using scanning tunneling microscopy and cantilever technology. In addition, we will discuss novel concepts in recent research including the use of achiral reagents for chiral sensing with NMR, and mechanical control of chiral sensing. The importance of integration of chiral sensing systems with rapidly developing nanotechnology and nanomaterials is also emphasized.

## 1. Introduction

Although various materials and devices have been fabricated, most paradigms of ‘intelligent’ systems are of biological origin. Perhaps surprisingly, they are simply self-assembled aggregates of functional molecules, which can be recognized as some of the ultimate products of supramolecular chemistry especially considering research fields concerning with self-assemblies [1–5]. One of the notable and interesting differences between biological and artificial systems is the fact that materials composing the former largely consist of chiral molecules. Therefore, while most biochemical processes routinely discriminate chiral molecules, differentiation between chiral molecules in artificial systems is currently one of the most challenging subjects in the field of molecular recognition [6]. It is well known that molecules of different chiralities (*i.e.*, enantiomers) can have significantly differing biological effects when, for instance, administered as pharmaceuticals. Often only one enantiomer of a chiral drug exhibits useful therapeutic effects while use of others may entail the risk of serious detrimental effects, so administration of single enantiomer drugs is recommended over the racemic alternative which has typically been used until fairly recently [7]. Thus, molecular recognition in biological systems is much more sensitive to the chirality of a substance than their artificial counterparts. One of the most ‘intelligent’ sensors could be a system capable of discriminating between molecules based on their chirality.

Of course, chiral sensing and recognition have been attractive and important targets in analytical chemistry. Chiral enantiomers have identical chemical formulae, molecular weight, and physicochemical properties with a few exceptions. Optical rotation has been used as a standard parameter to differentiate chiral molecules for some time [8]. Although optical rotation is an essential parameter of chiral compounds, several problems arise during its evaluation: (i) optical properties do not always have a simple relation to the enantiomeric purities; (ii) optical rotation at 100% purity is often unknown and that makes calculation of optical purity inconvenient; (iii) contamination of chiral compounds often makes the data complicated; (iv) precise setting of measurement conditions including temperature, concentration, solvents, and measuring wavelength is required. Therefore, alternative methods of chiral analysis have been pursued. One of the most common methods to evaluate molecular chirality is circular dichroism (CD) spectroscopy [9]. Even when target molecules do not have strong absorption peaks, chiral information can be transmitted to interacting molecules with a large extinction coefficient, resulting in induced CD (ICD). Nuclear magnetic resonance (NMR) spectroscopy is also a powerful technique for providing detailed information on molecular structure. Molecular interaction and/or direct covalent bonding between target molecules and chiral additives can lead to split resonances and/or variation in chemical shift [10,11]. Initially, it was believed that such additives must be chiral in nature, but this intuitive view has been disproved by recent research on the subject (see later in this review).

Strategies based on analyses after separation of chiral components are also often used. Mixtures of chiral molecules can be resolved to separate chiral components by chiral column chromatography [12,13]. Analysis of their chromatograms provides information on enantiomeric purity and absolute configuration. For chiral column chromatography, polysaccharide-based phases such as cellulose esters as well as phenylcarbamates of cellulose and amylose are some of the most popular separations media. Since Louis Pasteur demonstrated spontaneous separate crystallization of tartaric acid from racemic mixtures to enantiomerically pure crystals, crystallization has become a useful method for separating chiral components. X-ray crystallographic analyses can be used to assign absolute configuration of an optically pure compound [14].

Although various methodologies on chiral sensing and resolution have been proposed, investigated, and even established, easier and more convenient techniques are always sought. Rather than use of column chromatography and/or X-ray crystallography, chiral detection by simple mixing in solution has much higher potential in practical use, especially in *in situ* evaluation of biological systems. Since intermolecular interactions and detection at surfaces are part of the scope of supramolecular chemistry [15–19] and interfacial sciences [20,21], chiral sensing based on those concepts is becoming more and more important. In this review, we briefly summarize recent advances in these fields and also introduce unusual challenges in chiral sensing.

## 2. Supramolecular Approaches

Because chiral recognition is an attractive and challenging research target in host-guest chemistry, various host molecules capable of discriminating chiral guests have been developed and are still hot topics in supramolecular design [22]. For example, Aida and coworkers synthesized a chromophoric cyclic host consisting of two zinc porphyrin units that are connected by oligo (aminoisobutyric acid) posts [23]. The latter post units are in an equilibrium mixture of thermodynamically interconverting right- and left-handed helices and only inclusion of helical guests induces intense chiroptical signals. The same research group reported a novel heterocyclic porphyrin dimer containing an asymmetrically distorted *N*-alkylporphyrin as the first host molecule capable of sensing chiral fullerene C_76_ by means of ^1^H-NMR spectroscopy [24]. Kim, Inoue, and coworkers used achiral molecular cucurbiturils with significant enantiomeric and diastereomeric discrimination by incorporating a strong chiral binder [25]. (*S*)-2-methylbutylamine as the strong binder was discriminated by two enantiomeric supramolecular hosts, composed of cucurbituril[6] and (*R*)- or (*S*)-2-methylpiperazine. Borhan and coworkers investigated a porphyrin tweezer host with which chiral substrates exhibited exciton-coupled bisignate CD spectra with predictable signs [26]. Absolute configurations of a variety of *erythro* and *threo* guests could be clearly determined. Suzuki and coworkers synthesized a secondary terephthalamide host attached to four aryl blades [27]. A conformational change from a nonpropeller anti-form to a propeller-shaped syn-form upon complexation with ditopic guests results in much stronger chiroptical signals (chiroptical enhancement). Recently, Nakashima *et al*. reported optical activity and chiral memory effect thiol-capped CdTe nanocrystals by the ligand exchange of chiral components with an achiral thiol [28].

Color detection on chiral sensing would be a most convenient monitoring system useful for *in situ* chiral examination, and would contribute greatly to pharmaceutical research fields. When converting chiral recognition phenomenon into a change of color, the design of the host molecule attached to the chromophore is critical. Outstanding and pioneering work was performed by Kubo and coworkers who developed a calixarene host carrying two indophenol dye moieties and a binaphthyl group [29]. When a guest molecule such as phenylglycinol was added to the host dissolved in ethanol, the solution color changes depending on the chirality of the guest. The original color of the guest-free host is red, but addition of (*R*)-phenylglycinol causes a change in color to blue-purple due to a bathochromic shift of the indophenol absorption band (515.5 to 538 nm) together with the appearance of a new band at 652.5 nm. Interaction between the host binaphthyl group and the guest phenyl group induces variation in the hydrophobic environment about one of the indophenol dye moieties with deprotonation of the other indophenol group. In contrast, the solution color remains red upon the addition of (*S*)-phenylglycinol. Binding (*S*)-phenylglycinol to the host produces a complex with different relative positions of the phenyl and binaphthyl groups, resulting in suppression of the spectral shift of the dye moieties and a less pronounced color changes.

James and coworkers developed improved fluorescent chiral discriminating systems where a binol-based bisboronic acid host was used for enantioselective binding of a range of saccharic acids with a chiral sensitive fluorescence response [30]. This system is expected to be useful for the analysis of metabolic intermediates. Mei and Wolf developed a C_2_-symmetric sensor molecule, 1,8-bis(3,3′-(3,5-dimethylphenyl)-9,9′-diacridyl) naphthalene that underwent stereoselective interactions with a variety of chiral carboxylic acids, resulting in fluorescence quenching [31]. Tsukube and coworkers developed cholesterol-armed cyclens that can work as octadentate receptors for Na^+^, Ca^2+^, and Y^3+^ complexes [32]. The resulting helical metal complexes exhibited unique amphiphilic properties and provided chiral self-aggregates in aqueous solutions. Various dansylamino acid derivatives could be accommodated in the helicate aggregates to give highly enhanced fluorescence signals. Fuji and coworkers developed optically active artificial host molecules based on a phenolphthalein skeleton for the visual enantiomeric recognition of alanine derivatives [33].

**Figure 1.**
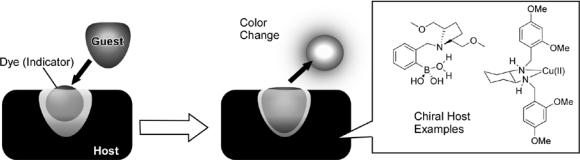
Indicator-displacement assay.

Syntheses of host molecular sensors co-possessing recognition sites and color-indicator moieties in sophisticated design are not always easy. These two roles can be supramolecularly assembled, which was realized as the concept of indicator-displacement assay [34] (Figure 1). This involves the use of colorimetric or fluorescent indicators that change optical or electrochemical properties when bound to a host through various effects such as fluorescence resonance energy transfer or photoinduced electron transfer with the host. Binding of a guest to the host liberates the indicator to the external medium yielding different spectroscopic characteristics. The guest binding can be detected by color changes, even though the host does not possess dye moieties. A similar concept was initiated by Inoue and coworkers [35] and Shinkai who used a guest labeled with an indicator [36]. Upon addition of an unlabeled analyte, displacement of the labeled analyte occurred inducing a color change. Anslyn and coworkers have popularized the indicator-displacement assay through a widely recognized standard method for sensor design [37]. In particular, they developed host molecules that can recognize polycarboxylic acids in foods and drinks using such an assay. Estimation of aging periods of Scotch whisky and Pinot Noir wine were demonstrated [38,39]. Use of hosts with chiral sensing capability (Figure 1) for indicator-displacement assay enables us to detect chiral guests through color changes [40,41]. Indicators can be selected to cover the largest dynamic range in absorbance or emission modulations for target guests. Extensive algebra allows one to derive a polynomial that relates enantiomeric excess to absorbance values.

Differences in supramolecular interactions can be converted to differences in the kinetics of chemical reactions and the products can be monitored by mass spectroscopy. Siuzdak, Finn, and coworkers use this concept for quantitative analyses of enantiomeric excess of chiral amines and alcohols [42]. Condensation of chiral alcohol guests with carboxylic acids with a chiral center results in diastereomeric products with different reaction speeds. If the carboxylic acid pair used has a difference in molecular weight, the resulting diastereomeric products can be directly monitored by mass spectroscopy giving values of enantiomeric excess of the guests. They demonstrated that a ratio of only 1.2 between two reaction rates makes it possible to quantify enantiomeric excess. This means that only small differences (*ca.* 0.1 kcal mol^−1^) in activation energy between the diastereomer formation is sufficient for reliable analyses of the enantiomeric excess. Such high throughput analysis by mass spectroscopy is especially useful for determining large numbers of samples. Therefore, this method could contribute significantly to exploration of chiral catalysts in combinatorial approaches. Strategies based on mass spectroscopy are widely used as practical methods for chiral sensing, and thus many examples have been reported. In particular, mass spectroscopy can analyze supramolecular interactions of chiral components in gas phases where perturbation by solvation can be avoided. Cooks and coworkers used the kinetics of competitive unimolecular fragmentations of trimeric Cu(II)-bound complexes for enantiomeric analysis of d,l-amino acids [43]. The same research groups demonstrated enantiomeric quantification of peptides by mass spectrometry [44]. The chiral analysis was performed on the basis of two parallel ion/molecule reactions followed by low-energy dissociations.

**Figure 2.**
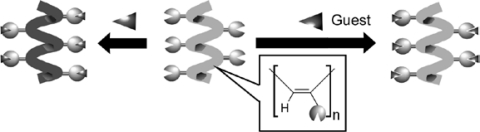
Helicity inversion based on guest binding.

For development of chiral sensing materials, design and preparation of polymeric materials with molecular sensing capability are crucial. Typical successful examples can be seen in a series of research on dynamic helical polymers such as poly (phenylacetylene) by Yashima and coworkers [45]. In these polymers, interaction at the side chain with chiral guests often occurs cooperatively with enhancement of chiral effects inducing twisting of the polymer main chains accompanied by strong ICD. Such effects result in highly sensitive detection of chiral substances and this method could be used for determination of absolute configuration and quantification of enantiomeric excess. For example, they synthesized a stereoregular (*cis*-*transoidal*), chromophoric poly(phenylacetylene) having a bulky *β*-cyclodextrin residue as the side group with molecular recognition capability (Figure 2) [46]. This polymer exhibited a color change (from yellow-orange to red) with a negative Cotton effect in the presence of excess (*S*)-1-phenylethylamine. In contrast, the polymer solution remained yellow with a positive Cotton effect upon addition of (*R*)-1-phenylethylamine. In another trial, a synthetic hydrochloride of poly (4-(*N*,*N*-diisopropylaminomethyl)phenylacetylene) exhibited a unique hierarchical amplification of chiral information from a non-racemic guest to macromolecular helicity in the liquid crystalline phase [47]. Upon complexation with an oppositely charged non-racemic acid as a dopant through electrostatic interaction in dilute water, the macromolecular helicity was further amplified in the polymer backbone as a greater excess of a single-handed helix through self-assembly into a lyotropic cholesteric liquid crystal. In other research, liquid crystalline poly (phenylacetylene) bearing ethyl phosphonate pendant groups can be used for chiral sensing of chiral pyrrolidines and piperazines [48]. Similarly *cis*-*transoidal* poly (phenylacetylene) bearing strongly acidic functional groups as pendants, such as a phosphonic acid or its monoethyl ester, or a sulfonic acid, can be used for sensing of various biomolecules such as peptides, proteins, amino sugars, and carbohydrates in water [49]. They also developed water-soluble oligoresorcinols for control of helical structures through addition of water-soluble chiral compounds in water at pH > 7 [50].

Structurally well designed polymers show enhanced optical properties that can be used for sensitive detection of chiral guests. For example, Pu and coworkers synthesized dendrimers containing a 1,1′-binaphthyl core and cross-conjugated phenylene dendrons [51]. The fluorescence intensity from these dendrimers can be efficiently quenched by chiral amino alcohols in an enantioselective manner. The higher generation dendrimer in particular is more sensitive to chiral quenchers due to enhanced energy migration and light harvesting effects. Lin and coworkers developed 1,1′-binaphthyl-based oligomers linked through their 6,6′-positions, ranging from quaternaphthol to decanaphthol [52]. The synthesized oligomers showed enhanced fluorescence as the chain length increases. For example, fluorescence intensity of oligonaphthols is almost two orders of magnitude higher than that of pristine 1,1′-bi-2-naphthol. They can be used for efficient enantioselective sensing through quenching with *trans*-1, 2-diaminocyclohexane resulting in an enantioselectivity factor of 1.24.

Imprinting of molecular forms into polymeric matrices may be also a good strategy for sensing particular shapes of guest molecules [53–55]. Cavities memorizing functional groups and the precise configuration of chiral guest molecules can be created in these matrices. Li and coworkers combined the concept of molecular imprinting with photonic porous polymers for colorimetric detection of chiral molecules [56]. Macroporous hydrogel was first synthesized through polymerization using silica colloid as a template in the presence of l-DOPA (3,4-dihydroxy-l-phenylalanine). The synthetic hydrogel displays a greenish color based on the porous structure, which can be hypochromically shifted to a blue color upon addition of l-DOPA. Binding of l-DOPA to the imprinted gel induces shrinking of the gel resulting in a change of color. In contrast, d-DOPA does not cause a color change because of its weak binding to the imprinted gel. Similarly imprinted polymers have been used for chiral sensing. Levon and coworkers used a self-assembled monolayer (SAM) polymerized on an electrode surface [57]. An octadecylsiloxane layer was covalently bound onto an indium tin oxide (ITO) electrode surface in the presence of the chiral *N*-carbobenzoxyaspartic acid molecules. The thus-prepared sensors exhibited recognition properties toward one isomer of racemic *N*-carbobenzoxy-aspartic acids. Marx and coworkers used imprinted sol-gel films spin-coated onto an ITO electrode for chiral selectivity recognition of (*R*)- and (*S*)-*N*,*N*’-dimethylferrocenylethylamine based on electrochemical measurement [58]. The imprinted films were able to detect *ca.* 2 ppm of the target molecule, with very good enantioselectivity and low nonspecific adsorption. Imprinted films have been widely combined with various devices. Willner and coworkers immobilized an acrylamide-acrylamidephenylboronic acid copolymer membrane on a quartz crystal microbalance (QCM) and on the gate surface of an ion-sensitive field effect transistor (ISFET) for nucleotide and monosaccharide [59]. Kharitonov and coworkers used the surface plasmon resonance (SPR) method for detection of nicotinamide adenine dinucleotide (NAD) and nicotinamide adenine dinucleotide phosphate (NADP) using cross-linked films consisting of the acrylamide-acrylamidophenylboronic acid copolymer [60].

## 3. Interfacial Techniques

As illustrated in the previous section, use of interfacial supramolecular phenomena between materials and devices such as QCM, functional electrodes, field effect transistor (FET), and SPR is crucial for advanced intelligent sensing. Such interfacial techniques between materials and devices result in various points of advantage for chiral sensing. For example, combination of advanced detection devices with rather simple chiral recognition can lead to highly enantioselective sensing systems, accompanied with a finer detection limit and more precise quantification. Techniques to immobilize materials at an interface provide dense oriented arrays of recognition sites as seen in thin film immobilization by SAM [61–64], Langmuir-Blodgett (LB) films [65,66], and layer-by-layer (LbL) adsorption [67–70]. These structures should enhance enantioselectivity through cooperative recognition. Interfacial recognition makes multiple screening possible and can be a good mimic of surface recognition at biomembranes.

**Figure 3.**
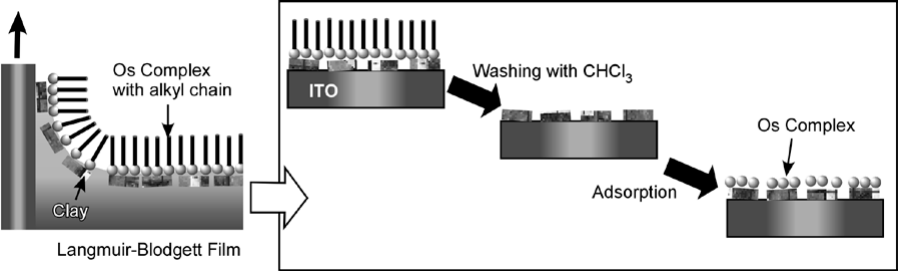
Modification of ITO electrode with Os complex by LB and SAM methods.

Electrochemical sensing at an electrode surface is one of the most widely used methods. In order to convert chemical chiral sensing to electrical signals, Yamagishi and coworkers prepared ITO electrodes with clay composite and a metal complex, Λ-[Os(phen)_3_]^2+^ (phen = 1,10-phenanthroline) by combined methods of LB and SAM (Figure 3) [71]. With this modified electrode, chiral sensing of binaphthtol can be achieved through monitoring photo currents, where enantioselective oxidation of 1,1′-2-binaphthol can be mediated with the Os^II^/Os^III^ redox pair with an oxidation rate ratio of 1.8 between (*S*)- and (*R*)-enantiomers. Nakanishi, Osaka, and coworkers prepared SAM structures of homocysteine (Hcy) on a Au(111) surface for chiral recognition of DOPA through electrochemical detection using cyclic voltammometry (CV). Oxidation reaction depended significantly on the combination of Hcy and DOPA. Oxidation reaction proceeds for d-DOPA at l-Hcy SAM and l-DOPA at d-Hcy, while DOPA is hardly oxidized with the homochiral combination (d-Hcy+d-DOPA or l-Hcy+l-DOPA). For example, the l-Hcy SAM facilitates permeation of d-DOPA to the electrode surface but suppresses permeation of l-DOPA, resulting in selective electrochemical detection on d-DOPA [72]. They also used the same SAM on electrodes for the detection of other amino acids such as enantiomers of alanine and leucine, where CV for the deposition of Cu from Cu complexes of the amino acids at an Au electrode modified with Hcy SAM [73] was monitored. Switzer *et al*. demonstrated that chiral surfaces can be produced through electrodeposition and that organic molecules adsorbed on surfaces have profound effects on the morphology of the inorganic deposits [74]. Electrodeposition of a copper oxide film on an achiral gold surface in the presence of tartrate indicated that the chirality of the ion determines the chirality of the deposited film.

Hierlemann and coworkers demonstrated a combined sensor that can quantify gas samples such as chiral amino acid derivatives and lactate with high sensitivity in real-time analyses [75]. A QCM sensor coated with chiral polymers can provide the difference of surface affinify of the chiral analytes from frequency changes. A partner sensor uses reflectometric interference spectroscopy (RIfS) that can measure changes in optical thickness upon chiral gas deposition to chiral matrix polymers. These methods are superior from the viewpoint of repeated use. In addition, high sensitivity for easy detection of 10% ee differences was achieved. In particular, the QCM technique has been more popularly used for chiral sensing, because this method can detect nanogram or subnanogram quantities of adsorbed materials in both gas phases [76] and solution phases [77]. Mass-sensing mechanisms can be applied to many kinds of targets. Cheng and coworkers used dicyclodipeptide-bearing calix[4]arenes immobilized on a QCM surface for enantiomeric recognition of (*R*)-methyl lactate [78]. Paolesse and coworkers synthesized a Co complex of chiral porphyrin diads that was self-assembled on QCM electrodes to be used for nanogravimetric sensors with enantiodiscrimination in the gas phase [79]. Osaka and coworkers reported QCM sensing for the chiral amino acids d- and l-phenylalanine based on enantioselective adsorption onto (*R*)- and (*S*)-1,1′-binaphthalene-2,2′-dithiol SAM structures on gold electrodes [80]. Xu *et al*. developed chiral sensors based on the self-assembly of perfunctionalized *β*-cyclodextrins on a QCM sensor for real time chiral recognition of enantiomeric alcohols and lactates [81]. Eun and Umezawa applied QCM sensing for l-leucine sensing based on growth of l-leucine crystals immobilized on a monolayer of 11-mercaptoundecanoic acid [82]. Kim and coworkers demonstrated highly selective recognition of chiral mandelic acid using l-phenylalanine as the selector [83]. Their method is based on QCM detection, integrated with a vapor diffused molecular assembly reaction technique. Toyooka and coworkers applied QCM sensing for prediction of the separation efficiency of a pair of enantiomers during chiral high-performance liquid chromatography (HPLC) [84]. When chiral separation is possible using a chiral stationary phase immobilized on the sensors, significant differences in the frequency changes are observed because the intensities based on interactions differ among the isomers. Willner coworkers combined the QCM method with Faradaic impedance spectroscopy, chronopotentiometry, and SPR for characterization on the swelling of acrylamidophenylboronic acid-acrylamide hydrogels upon interaction with chiral glucose [85].

SPR is another popular technique for surface sensing in detection of adsorption of external guests. Koh and corworkers used the SPR method for phenylalanine sensing using a photochromic spiroxazine derivative [86]. The different SPR angle shift derived from interaction between d- and l-phenylalanine and spiroxazine monolayer can be explained by the different dipole moment of the ionic complexes. Recently, Markovich and coworkers reported combining plasmon-enhanced absorption with CD [87]. CD signals were enhanced by two orders of magnitude which was a result of comparable enhancement of the overall electronic absorption of specific probe molecules on colloidal silver nanoparticles. Enhanced sensitivity of CD signals are useful especially for investigation on conformations of biological molecules and probing samples at lower concentration. Katz and coworkers similarly discussed combining CD and SPR mechanisms [88]. They used gold nanoparticles post-synthetically modified with chiral 1,3-disubstituted diamino calix[4]arene ligands that exhibit a CD-active SPR absorption band. It is based upon the influence of the asymmetric center of the chiral adsorbate on the electronic states of the metal nanoparticle core and an explanation supported by the observed interactions between the gold surface and adsorbed ligand.

**Figure 4.**
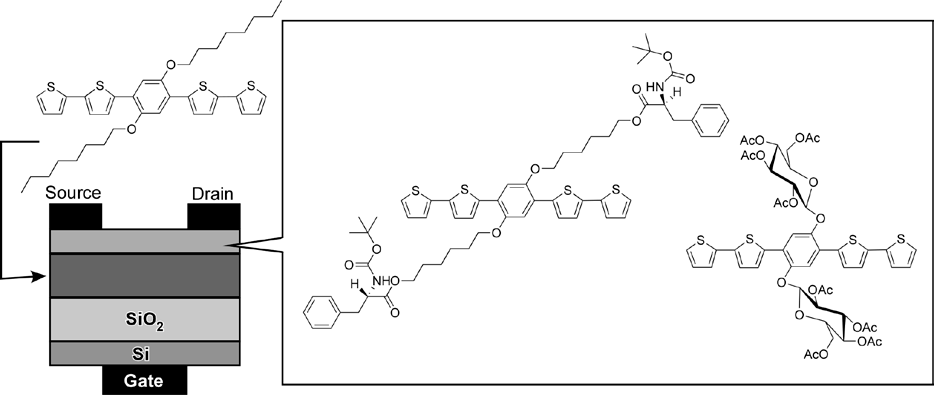
Layer-type FET sensor with alkoxythiophene oligomer with chiral recognition sites.

Attention has been also paid to thin film transistors as another sensing device in chemical and biological sensing [89–93], because they can have high potential to integrate into more advanced device systems. Improvements on selectivity and sensitivity have been paid much attention. For chiral sensing, conducting-polymer solid-state chiral detection so far provides high sensitivity. Recently, Torsi improved sensitivity to ppm level in enantiomeric selectivity using a layer-type FET sensor with chiral components (Figure 4) [94]. This FET sensor incorporates an alkoxythiophene oligomer with chiral recognition sites for saccharides and amino acids at an external side and an alkoxythiophene oligomer with octyl group at a gate side. This sensor provides both high sensitivity and easy usability.

Because biomolecules usually possess chiral structures and often possess superior chiral sensing capability, integration of biocomponents into sensing systems is a very promising idea to produce intelligent chiral sensing systems. For example, the use of antigen-antibody processes has sometimes resulted in highly sensitive chiral sensing. Green and coworkers first immobilized streptavidin to a gold surface *via* dexrtran linkage and then fixed a d-phenylalanine derivative through avidin-biotin interaction [95]. Binding of the antibody for the d-phenylalanine derivative on the modified surface causes sensitive changes of surface plasmon response. Using this mechanism, trace amounts of the d-phenylalanine derivative contaminated with the l-analogue can be quantified with high sensitivity. For example, 0.1 μM of d-amino acid can be detected in the presence of 250 μM of l-amino acid. This sensitivity is 10 times higher than those of the conventional HPLC analyses. In addition, high reusability (up to 100 times) was confirmed. Josephson and coworkers developed a highly sensitive chiral sensing system by combining magnetic resonance imaging (MRI) and immunoassay [96]. They modified superparamagnetic iron oxide nanoparicles with d-phenylalanine and cross-linked dextran. Addition of the antibody for d-phenylalanine to a dispersion of this nanoparticle induced aggregation of the nanoparticles, resulting in a decrease in the T2 relaxation time of water by 100 ms. Because the presence of unbound d-phenylalanine significantly suppresses the nanoparticle aggregation accompanying with increase of T2 value, the amount of d-phenylalanine contaminant in l-phenylalanine can be sensitively detected. NMR measurement of the T2 parameter makes it possible to detect 0.1 μM of d-amino acid in the presence of 10 mM of l-amino acid (99.998% ee). Upon application of this system to MRI, high throughput ability (measurement of 60 samples within 2 minutes) can be achieved. Availability of a wide range of chiral targets can be realized by just changing selectivity of the antibodies. Other chiral sensing systems combined with biomolecules have also been investigated. Ng and coworkers demonstrated real-time chiral discrimination of enantiomers of 3-methoxyphenylethylamine, tetrahydronaphthylamine, 2-octanol, and methyl lactate using a bovine serum albumin (BSA) or human serum albumin (HSA) functionalized QCM biosensor [97]. Rotello and coworkers reported protein recognition with amino acid and dipeptide-functionalized gold nanoparticles [98]. Fornstedt and coworkers compared the SPR assay and the HPLC perturbation method for drug-protein interactions and pointed out risks on considerable quantitative deviations in some cases [99].

Recent surprising advancements on probe microscopies enable us to directly see actual molecules [100,101]. Direct visual discrimination of chiral molecules becomes possible. For example, Lopinski and coworkers differentiate *cis*-2-butene and *trans*-2-butene adsorbed on Si(100) surface using scanning tunneling microscopy (STM) [102]. When *trans*-2-butene is adsorbed on the Si(100) surface, bond formation with a silicon dimer results in the creation of (*SS*) and (*RR*) enantiomers. Orientation of the methyl group can specify enantiomers under STM observation. Because the *cis* isomer does not produce enantiomers, estimation of the *trans* and *cis* isomer ratio can be also done on the basis of direct observation. Another impressive advanced technology of nano-mechanisms would be cantilever technology [103,104] that can be also used for chiral sensing. For example, Hofstetter, Sepaniak, and coworkers demonstrated chiral detection through deformation of a cantilever [105]. They modified a gold surface of the cantilever with 2-aminoethanethiol where an anti-L-amino acid antibody was further immobilized via cross-linking with glutaraldehyde. Addition of L-amino acid caused significant deformation while the d-analogue did not show deformation of the cantilever. No deviation of the deformation values was observed even when 200 mg L^−1^ of d-amino acid was added to 0.2 mg L^−1^ of the l-analogue (99.8% ee). In addition, the deformation degree per time is in good linear relation with concentration of amino acids in range from 0.2 to 100 mg L^−1^.

## 4. Unusual Challenges

In the final section, several unusual approaches will be briefly introduced. Direct sensing of chiral molecules needs specialized spectroscopic methods such as optical rotation and CD. If we use other methodologies such as NMR, we have to add chiral additives to differentiate chiral analytes. However, the potential of analytical methods for chiral sensing would be expanded greatly if chiral signaling using non-chiral additives could be realized. Creating chiral signals from achiral (non-chiral) components is undoubtedly a major challenge.

**Figure 5.**
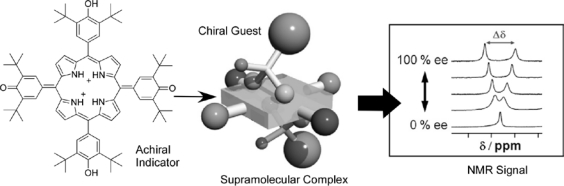
Quantification of enantiomeric excess of chiral guests by nonchiroptical spectroscopy, NMR, with achiral additives through supramolecular complex formation.

Fujita and coworkers reported an increase in enantiomeric purity of binaphthol using an achiral supramolecular Pd-coordinated capsule [106]. Their capsules can accommodate a pair of (*R*) and (*S*)-binaphthol molecules and this inclusion can increase enantiomeric purity of the binaphthol remaining in solution. For example, naphthol in 50% ee was converted to 80% ee. This example is not a sensing system but suggests the usefulness of achiral supramolecular complexes for chirality control. Very recently, we have demonstrated quantification of enantiomeric excess of chiral guests such as mandelic acid by a nonchiroptical method, NMR spectroscopy with achiral additives, through supramolecular complex formation (Figure 5) [107]. Addition of pure chiral mandelic acid to a dueterated chloroform solution of an achiral porphyrin derivative induces peak splitting of the *β*-proton resonance of the porphyrin core and *ortho*-proton resonance in the phenyl ring. Decreasing enantiomeric purity of the mandelic acid guest suppresses the peak splitting. Critically, splitting width makes a good linear relation with enantiomeric excess of mandelic acid, enabling us to calculate enantiomeric excess from NMR signals of achiral molecules. Established methods of NMR chiral sensing rely on the use of chiral derivatizing reagents (covalent or salt forming type) and/or chiral solvating reagent (non-covalent type), which can convert chiral guests to diastereomers with deviation of chemical shifts and peak integral area. Therefore, it was believed that chiral derivatizing reagents and chiral solving reagents must be chiral. However, the abovementioned example disproves this intuitive view and possesses a great potential for expanding the use of achiral reagents for chiral sensing using NMR spectroscopy.

**Figure 6.**
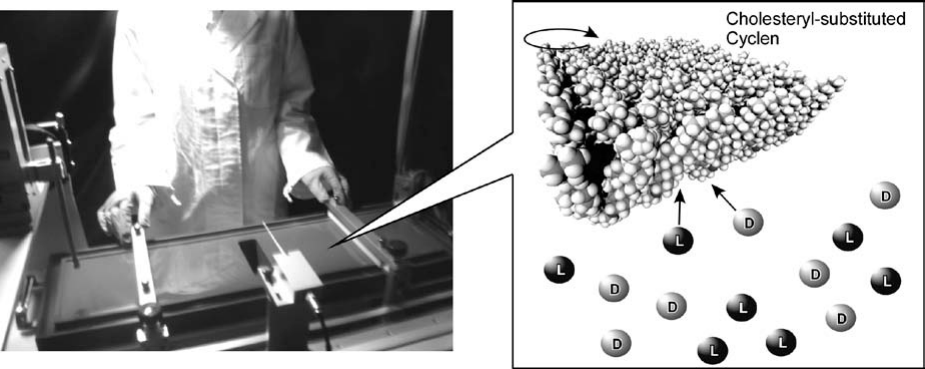
Mechanical control of chiral guest binding to monolayer of cholesteryl-substituted cyclen

As demonstrated experimentally [108,109] and theoretically [110,111], Langmuir monolayers at the air water interface provide appropriate media for molecular recognition for various aqueous guest molecules including chiral biomolecules such as peptides [112–114]. Chiral recognition at the air-water interface basically depends on molecular design of monolayer components as seen in amino acid recognition with amphiphilic chiral crown ether monolayers by Rogalska and coworkers [115], enantioselective recognition of phenylalanine on a monolayer of a metal complex of chiral amphiphilic calyx [4] resorcinarene by Shahgaldian *et al*. [116], and chiral induction by achiral barbituric acid derivatives by Liu and coworkers [117]. Unlike these previous examples, we pioneered control of chiral recognition by mechanical force application at the air-water interface. We previously developed controllable guest capture and release using a steroid cyclophane under application of mechanical forces to its monolayer [118,119]. Recently, this concept of controllable molecular recognition has been expanded to chiral recognition using a cholesteryl-substituted cyclen complex host molecule (Figure 6) [120]. The octacoordinate sodium complex of the cholesteryl-substituted cyclen has two possible quadruple helicate structures. Helicity is influenced by the chirality of the side arms, especially when ordered or aggregated at the supramolecular level. Therefore, sensing of chiral guest molecules at the hydrophobic cavities also affects the helicity. Binding of aqueous amino acids in the aqueous subphase of the monolayer was examined based on changes in isotherms between surface pressure and molecular area. In the case of leucine sensing, binding constants of d-leucine are always greater than those of l-leucine at all the surface pressure ranges investigated, indicating that the monolayer of cholesteryl-substituted cyclen has a stronger interaction with d-leucine. Interestingly, enatioselective binding of valine to the same monolayer is highly affected by surface pressure. The binding constant values of l-valine are smaller than those of d-valine at low surface pressure but exceed them at 22–23 mN m^−1^. This means that chiral recognition in the monolayers of cholesteryl-substituted cyclen with valine changes from the d- to l-form upon compression. This is a clear example of tuning of chiral discrimination by bulk mechanical force. The latter experimental results are a very unusual example in chiral sensing, because we can freely tune chiral selectivity just by changing the compression state of the monolayer. Actually, molecular compression was done using a film balance machine, but the same concept can, in theory, be reproduced by film compression by our hands. Therefore, this system can be regarded as hand-tuned chiral sensing, which is a part of our new concept hand-operated nanotechnology [121].

## 5. Future Perspectives

Chiral sensing is one of the most challenging targets in analytical chemistry due to the necessity for differentiation of very slight differences in molecular structure. At the same time, this target is very important for future technology because most important chiral substances are bio-related molecules and their sensing contributes to biology, biotechnology, and pharmacy. Chiral sensing processes consist of two processes, molecular sensing and signal transduction. If we more closely consider intelligent chiral sensors, inclusion of concepts on nanotechnology and nanomaterials becomes more important [122–125]. Although the details cannot be here described, typical examples are listed below with their references for further consideration. There have been great advances in nanostructure fabrications and some of these efforts have successfully created novel, useful concepts such as the atomic switch [126,127], probe-fabrication of molecular arrays [128–130], and integrated circuit technology [129]. In addition, various nanostructures have now become available such as carbon nanotubes [131–136] and other nanotubes [137–140], nanosheets [141–144], nanoparticles [145–149], nanorods [150–153], nanowires [154–156], nanowhiskers [157–159], mesoporous silica [160–162], mesoporoous carbon [163–165] and other mesoporous materials [166–168], organic-inorganic nanohybrids [169–171] and bio-related nanohybrids [172–174]. Although these nanostrctures and nanomaterials have surprising structural precision, ordering and orientation, very high surface areas and other textural parameters [175–177], and high functions such as electronic [178–179], photonic [180–182], magnetic [183–187], and catalytic properties [188–190], they have not been used as chiral sensors. Important developments of intelligent chiral sensors will likely be achieved through integration of chiral sensing units and concepts described in this review as well as through the integration of advanced nanostructures and nanomaterials.
